# Innovation contests to promote sexual health in china: a qualitative evaluation

**DOI:** 10.1186/s12889-016-4006-9

**Published:** 2017-01-14

**Authors:** Wei Zhang, David Schaffer, Lai Sze Tso, Songyuan Tang, Weiming Tang, Shujie Huang, Bin Yang, Joseph D. Tucker

**Affiliations:** 1 University of North Carolina Chapel Hill Project-China, No.2 Lujing Road, Guangzhou, 510095 China; 2 Guangdong Provincial Dermatology Hospital, No.2 Lujing Road, Guangzhou, Guangdong China

**Keywords:** Innovation contest, Sexual health, Community engagement, Youth, China

## Abstract

**Background:**

Innovation contests call on non-experts to help solve problems. While these contests have been used extensively in the private sector to increase engagement between organizations and clients, there is little data on the role of innovation contests to promote health campaigns. We implemented an innovation contest in China to increase sexual health awareness among youth and evaluated community engagement in the contest.

**Methods:**

The sexual health image contest consisted of an open call for sexual health images, contest promotion activities, judging of entries, and celebrating contributions. Contest promotion activities included in-person and social media feedback, classroom didactics, and community-driven activities. We conducted 19 semi-structured interviews with a purposive sample to ensure a range of participant scores, experts and non-expert participants, submitters and non-submitters. Transcripts of each interview were coded with Atlas.ti and evaluated by three reviewers.

**Results:**

We identified stages of community engagement in the contest which contributed to public health impact. Community engagement progressed across a continuum from passive, moderate, active, and finally strong engagement. Engagement was a dynamic process that appeared to have little relationship with formally submitting an image to the contest. Among non-expert participants, contest engagement increased knowledge, healthy attitudes, and empowered participants to share ideas about safe sex with others outside of the contest. Among experts who helped organize the contest, the process of implementing the contest fostered multi-sectoral collaboration and re-oriented public health leadership towards more patient-centered public health campaigns.

**Conclusion:**

The results of this study suggest that innovation contests may be a useful tool for public health promotion by enhancing community engagement and re-orienting health campaigns to make them more patient-centered.

## Background

Innovation contests can engage large groups of people in health-related activities [1]. For example, in August 2010 a research group in Seattle, USA, launched an innovation contest called *Foldit* [2]. The online multiple-person game allowed non-experts to play, compete, and collaborate. Within 12 months, the innovation contest had engaged nearly 60,000 participants. The founders then announced that the aggregate work of these gamers had uncovered the structure of a critical HIV retroviral protease, a problem that expert program developers had been unable to solve [2].

Innovation contests are a form of crowdsourcing that call on non-experts to solve problems, often enabled by multi-sectoral input from the community. Innovation contests have been used to promote public health in several contexts, including promoting HIV testing [3], encouraging condom use [4], identifying food deserts [5], improving sanitation [6], and shaping public health policy [7]. While most contest evaluations have focused on contest outputs, innovation contests may also promote community engagement. We define community engagement as the relationships and communication between local community members and health authorities [8]. Community engagement is critical for strengthening health systems [9]. A better understanding of community engagement can help to inform new intervention development and tailor existing interventions in order to increase local feasibility and acceptability.

China has a cultural tradition of crowd power that may facilitate the use of innovation contests in this region [10]. The Chinese government has identified crowdsourcing as a useful strategy for supporting innovation [11]. The Chinese State Council recently released guidance on building a platform to support innovation, specifically mentioning crowdsourcing in health.

Sexual health is an increasingly important topic in the Chinese context. Premarital sex is increasingly common and conventional sexual health education for youth is limited [10, 12, 13]. We organized an innovation contest in China with the goal of soliciting creative images to promote sexual health among Chinese youth. This innovation contest was focused on both generating useful images in addition to engaging the local community. The purpose of this qualitative research study was to describe the extent of community engagement in an innovation contest for sexual health promotion.

## Methods

This study took place in Guangzhou, China and was approved by the University of North Carolina Chapel Hill School of Medicine and Guangdong Provincial Dermatology Hospital institutional review boards. Informed consent was obtained from all individual participants included in the study. This study included the qualitative research presented in this manuscript and not the broader image contest. All potentially identifiable data was scrubbed of identifiers prior to data analysis. All data was encrypted in accordance with data handling procedures outlined by the UNC School of Medicine.

### Innovation contest

The innovation contest is described in detail elsewhere [14]. The contest was designed and implemented by our SESH (Social Entrepreneurship for Sexual Health) team that included public health authorities, CBO (community-based organization) leaders, and researchers. A series of community-based workshops and discussions among local partners [15, 16] informed the development of an open call for submissions. The call for entries clarified that the purpose was to solicit images promoting sexual health among young people. Then we organized a series of in-person events and social media activities to encourage submissions [14]. In-person activities focused on clarifying the rules of the contest, providing feedback on ideas, and avoided giving examples (to limit cognitive fixation). Individuals could submit more than one entry. Entries were evaluated with a score from 0 to 10 (0 = worst, 10 = best) by a group of 20 local judges including young people, researchers, public health experts, and artists. Judges identified three finalists who received prizes and additional prizes were given at an in-person event to coincide with local World AIDS Day events. During the final week of the contest, there was an online voting system so that individuals could vote for their favorites among the judge-determined top three entries.

### Participant recruitment

We conducted semi-structured interviews between March 2015 and June 2015. We used purposive sampling to ensure a range of participant scores (individuals who received high scores and low scores), experts and non-experts, those who submitted to the contest and those who did not. Experts were defined as medical or media professionals. We included individuals who either participated in the contest or helped to organize the contest. We made phone calls or emails to contact individuals and ask about whether or not they would be interested in joining the study. In order to understand implementation of the contest, we interviewed contest organizers (public health officials) and individuals at community-based organizations who helped organize in-person events. Three pretest interviews were conducted prior to the launch of the study to develop a standardized interview guide, which included questions regarding: expectations of participating in the contest, facilitators and barriers to contest participation, engagement in contest activities, and participants’ experience throughout the entire process of the contest.

Interviews were conducted by two qualitative research trained bilingual (Mandarin-English) researchers at a convenient, private location and time of participant’s choice. All interviews were conducted with only the interviewer and the participant. Follow-up interviews were conducted with three interview participants by phone in order to understand the durability of the effects of the contest over time. Each follow-up participant received one interview in order to better understand their knowledge level of sexual health, condom use, HIV testing habits and openness of talking about sex with peers. Written informed consent was obtained from each participant while verbal informed consent was obtained from participants in interviews over the phone. All interviews were recorded, transcribed, translated, encrypted and transferred into ATLAS.ti 7.0 qualitative research software.

### Qualitative data analysis

We used a qualitative approach to examine innovation contests for three reasons: 1) qualitative research provides an opportunity to examine the lived experience of local individuals who directly participated in the contest; 2) qualitative research can provide deep, rich information about contests that would not be captured in surveys; 3) qualitative data are important for the experience of community engagement. We used a Framework Approach [17] to identify potential themes. The framework was developed prior to the coding process, which was based on the interview guide. Two coders examined the primary data and drafted a codebook. A third individual later reviewed the coded texts and evaluated each transcript for consistency. When agreement of the codebook was reached, two coders used the finalized codebook and coded the remaining transcripts. We used a community engagement perspective in order to differentiate the depth and breadth of community engagement related to an innovation contest. Our community engagement perspective was informed by community-based participatory research principles [18].

## Results

Forty-three potential participants were contacted by email, text message and social media. Twenty individuals agreed to participate in our study. We conducted a total of 19 interviews among 20 individuals (one group interview). Our participants included mostly young, university students, contest participants, and living in Guangdong Province (Table 1). Students came from a variety of academic backgrounds, including fine arts, social science, media, pharmacy and medicine. Contest organizers included staff from HIV related community-based organizations and hospital staff who contributed to designing and implementing the contest.

**Table 1:** Demographic characteristics of interview participants in South China from interviews conducted in 2015 (*n* = 20)

	Number	Percent
Location		
Beijing	2	10%
Guangdong Province	18	90%
Age (years)		
18-24	12	60%
25-30	7	35%
31-35	1	5%
Gender		
Male	7	35%
Female	13	65%
Student status		
Student	13	65%
Non Student	7	35%
Engagement stage		
Participant	17	85%
Organizer	3	15%

### Community engagement in the contest

We observed a stage-wise progression of engagement in the contest over time (Table 2). We categorized participants’ level of engagement by examining their participation according to the following criteria: 1) gave comments on how to improve the contest; 2) extent of participation in in-person events and online promotion activities 3) shared contest information, ideas, or images with friends/families in-person or on social media; 4) intention to participate in future events; 5) helped organize the contest. We identified four stages of engagement, including passive, moderate, active, and strong (Table 2).

**Table 2:** Stage of South China sexual health contest engagement

Engagement stage	Passive	Moderate	Active	Strong
	(*n* = 3)	(*n* = 3)	(*n* = 8)	(*n* = 6)
Spectrum of Engagement				Helped with contest organization or promotion
			Intention to participate in future discussion and/or contests OR Helped with contest organization or promotion	Interested in participating in future discussion and/or contests
		Shared Ideas with friends and family on contest/image	Shared ideas with friends and family on sexual health/contest/image on social media	
	Single participation in contest activity^a^	Multiple participation in contest activities		
	Gave comments on contest improvement			
Selected quotes from participants	I went to the award ceremony on the World AIDS Day. (college student)	I shared with my colleagues when I finished the image, explained to them what I thought and they gave me some feedback. (HIV CBO staff, submitter)	(Name) told us this contest is concerning HIV/AIDS, so at the very beginning we were very proactive to help promote it. I think the contest was very creative and interesting. (Student Society member, non-submitter)	I retweeted contest information on my WeChat Moment. Also I introduced this contest to my classmates from college and grad school. (Image Contest organizer, submitter)

^a^Contest activities include following WeChat, voting for Image, participating in In-person events

Passive engagement indicated only participating in a single activity. Moderate participation indicated participation in multiple activities and sharing ideas with others. Active engagement indicated that participants had the intention to participate in future discussion and/or contest; helped with contest organization or promotion; participated in multiple contest activities and shared ideas with family. Strong contest engagement indicated that participants engaged in multiple contest activities; helped with contest organization or promotion, and were highly active in social media in terms of sharing contest or sexual health information. Submitting an entry to the contest was not significantly related to engagement. The goal of the contest was not to get entries from participants but to stimulate discussions and conversations on sexual health. We examined the contest participations among participants at different participation levels. Some participants who are at higher level of participation did not submit any entries while some submitters weren’t much involved in contest activities but submitted entries.

Three major themes were identified from participant data: (1) an increase in knowledge and healthy attitudes about sexual health; (2) multi-sectoral collaboration; and (3) a re-orientation of professionals towards patient-centered sexual health campaigns.

### Increased knowledge and healthy attitudes about sexual health

Contest participants reflected on pre-existing misconceptions of sexual health. The process of participating in in-person activities helped build positive attitudes and address these misconceptions: one medical student with strong engagement in the contest noted that while she was creating the image, she tried to build a connection with young people and address gaps in knowledge related to sexual health:

*“When I was creating the images for the contest, my teammate and I would think about how to convey our idea to the audience. So we tried to understand how our peers perceive sexual health and put that into our images. We hope we can deliver knowledge about sexual health and healthy attitudes about sex to people.”* (Medical student, 22, Strong engagement)

The contest aimed to promote sexual health in general, which covered a wide range of topic including sexuality, sexual attitude and behavior as well as sexually transmitted diseases such as HIV. One informant discussed his attitude about HIV testing in the follow up interview:

*“The image contest really got me to rethink the knowledge about sex that I had before. Although I get tested for HIV every year, now I would say I pay more attention to it because I received more knowledge about sexual health from the contest.”* (CBO Staff, 27, Moderate engagement)

During the contest, various activities were held to engage the public in which people would learn more about sexual health. Increased knowledge about sexual health enhanced the participants’ confidence talking about it with others. A contest organizer shared her idea:

*“By reading the posts promoting the contest on social media, I realized that there were things that I omitted [about sexual health]. With the experience helping promote the contest and knowledge that I got, I no longer think it is embarrassing to talk about sexual health with others.”* (Contest organizer, 26, Strong Engagement)

Considering sexual health related topics are rarely discussed in the public sphere in China, some people don’t think they have enough knowledge or are intimidated to talk about it with others. The contest has allowed people to learn more about sexual health and has created conversation among participants. Another informant noted:

*“I was very interested in raising public awareness on sexual health. I learned more about sexual health in order to help promote the contest, now I feel like I’m more capable and confident to talk about it.”* (Friend with CBO, 26, Moderate Engagement)

### Multi-sectoral Collaboration

We found that participation in contest events fostered communication among community-based organizations, public health authorities, and students. The contest spurred open conversations about sexual health and served as a platform for idea exchange. With participants who come from a range of backgrounds, the contest and its associated events served as a platform for networking. As described by one of our contest participants:

*“Through attending your events, I realized that you have connections with other organizations. It is a way to for you to understand other organizations and allow them to understand you.”* (Friend of CBO staff, 26, Moderate Engagement)

Contest events serve as a means of communication among the general population, community-based organizations and medical expects. An informant explained her experience participating in the World AIDS Day event:

*“In the ceremony, we invited experts, CBO members and people from the public to talk about sex. Some said they never had a chance to discuss sexual health before, like what parent education should look like, or even some questions that never occurred to them…this platform means communication. Experts giving talks conveys knowledge, but people with different backgrounds can give feedback to experts, for example their ideas on sexual health, or other topics related to sexual health. It is a way to raise awareness.”* (Contest organizer, 26, Strong Engagement)

### Re-orientation of public health campaigns

Some experts and contest organizers with strong contest engagement brought contest principles into their health work. This suggests that participation may have helped re-orient these health care providers towards more patient-centered public health campaigns. Public health campaigns are mainly led by professionals in China. Medical experts give advice to patients based on their own knowledge which might neglect the importance of patients’ feedback. By communicating with participants during the contest promotion, contest organizers could receive feedback from participants while spreading knowledge. A contest organizer recalled her experience organizing the events:

*“At first, people made jokes about it, asking, why are you working on STD (Sexually Transmitted Disease) activities. Later on, people started to understand my work, and paid more attention to [our] organization. Because we are all working in the public health sector and we’re sort of professionals, I can get a variety of feedback from them which would improve our work “*(Contest organizers, 26, Strong Engagement)

Motivated by the contest, some medical professionals demonstrate interest in applying innovation contests to other kinds of health campaigns:

*“I think it (the contest) can be applied to something else, not only for HIV/AIDS prevention but other health issues, for example, a smoking ban.”* (Medical Student, 22, Active Engagement)

## Discussion

Our qualitative evaluation of an innovation contest found benefits for both non-expert and expert participants. A wide range of community engagement was observed in the innovation contest and this engagement empowered participants to talk about sexual health. Additionally, the contest motivated experts to reconsider the design sexual health campaigns. Previous evaluations of innovation contests have mostly focused on optimizing outcomes [19, 20] and ignored the process of contributing and engagement [7]. Our research extends the literature by examining the process of organizing an innovation contest, differentiating stages of contest engagement, and examining how contest engagement may influence behaviors.

We found a broad range of community engagement that resulted from our contest. The broad range of community engagement is consistent with findings from an innovation contest in the United States designing images depicting locations of defibrillators [21]. This resonated with research that has been done on crowdsourcing contests in China on *zhubajie.com* and urban planning projects in US [10, 22]. This broad range suggests that the strength of community engagement cannot be measured by a single criteria alone. Participant engagement far exceeded our expectations and reached well beyond the most basic metrics that we evaluated. Many participants attended public events focused on discussing sexual health with peers, teachers, and others. Other participants initiated conversations about sexual health with friends and families, including via personal social media accounts.

We found varying stages of community engagement related to the innovation contest. Little research has examined engagement related to crowdsourcing projects [22, 23]. Yet, these research studies did not differentiate participation stages. We observed that participants moved through different stages of participation. Some participants were less involved in the contest when it began, but as our promotion proceeded, these individuals increased their engagement. However, others only had high engagement at the start of the contest. This underlines the importance of an open call for participation.

Contest engagement empowered the community to define what is important in sexual health. In the CrowdOutAIDS project – a campaign that used crowdsourcing to engage youth with topics in sexual health – individuals were able to take ownership over the project and determine its outcome [24]. Crowdsourcing can integrate the perspectives of the general population into stronger solutions than can be developed by a single individual. In the process of developing these solutions, participants make an effort to have their voices heard and learn from the ideas of others. During our interviews, participants were eager to share their ideas on the contest by providing reflections on their experiences and suggestions for future events. Participants indicated that our contest offered them a platform to talk about sexual health in an open setting and a chance to reflect on the topic, which they were rarely able to do before. To make this contest more visible, students affiliated with student societies endeavored to help organize in-person promotional events. Contest participants made great efforts to disseminate useful and positive information about sexual health by sharing ideas on how to create images with their friends and families. Furthermore, participants took advantage of WeChat, the most popular social media platform in China, when promoting the contest or their individual images. The generated discussion among contest participants goes beyond the “top-down” knowledge dissemination and encourages a “bottom-up” approach to public health.

Two key limitations should be noted. First, our promotion events targeted colleges, leading to a large number of well-educated participants. However, there is a group of higher education individuals with increased sexual risk in China [25]. Second, our interviews focused on behavioral sexual health outcomes and did not measure biomarkers or self-reported test uptake.

Our research has several policy and research implications. Many innovation contests have concentrated exclusively or disproportionately social media contributions, judging, and implementation [26]. Our innovation contest suggests the value of incorporating in-person events to promote community engagement. Second, open innovation contests cultivate collaboration amongst professionals of different fields [27]. Innovation contests represent a new tool for incorporating multi-sectoral input and may be useful for policy makers [28]. In terms of research priorities, further evaluation of innovation contests is needed to better understand their impact. Implementation science on how to optimally select non-experts and solicit contributions would be helpful.

## Conclusion

Innovation contests can lay a strong foundation for enhanced community engagement. They facilitate the collaboration of individuals with diverse backgrounds and skills to contribute to common goals. Through this process, a broad range of community engagement empowers the public to help shape health campaigns. Innovation contests provide a broad platform for participants to explore sexual health.

## Abbreviations

AIDS: Acquired immune deficiency syndrome
CBO: Community - Based Organization
HIV: Human immunodeficiency virus
STD: Sexually transmitted disease
STI: Sexually transmitted infection
